# An optimisation-based iterative approach for speckle tracking echocardiography

**DOI:** 10.1007/s11517-020-02142-8

**Published:** 2020-04-07

**Authors:** Neda Azarmehr, Xujiong Ye, Joseph D. Howes, Benjamin Docking, James P. Howard, Darrel P. Francis, Massoud Zolgharni

**Affiliations:** 1 School of Computer Science, University of Lincoln, Lincoln, UK; 2 National Heart and Lung Institute, Imperial College London, London, UK; 3 School of Computing and Engineering, University of West London, London, UK

**Keywords:** Strain imaging, Speckle tracking echocardiography, Myocardial deformation, Echocardiography

## Abstract

Speckle tracking is the most prominent technique used to estimate the regional movement of the heart based on echocardiograms. In this study, we propose an optimised-based block matching algorithm to perform speckle tracking iteratively. The proposed technique was evaluated using a publicly available synthetic echocardiographic dataset with known ground-truth from several major vendors and for healthy/ischaemic cases. The results were compared with the results from the classic (standard) two-dimensional block matching. The proposed method presented an average displacement error of 0.57 pixels, while classic block matching provided an average error of 1.15 pixels. When estimating the segmental/regional longitudinal strain in healthy cases, the proposed method, with an average of 0.32 ± 0.53, outperformed the classic counterpart, with an average of 3.43 ± 2.84. A similar superior performance was observed in ischaemic cases. This method does not require any additional ad hoc filtering process. Therefore, it can potentially help to reduce the variability in the strain measurements caused by various post-processing techniques applied by different implementations of the speckle tracking.

**Graphical Abstract:**
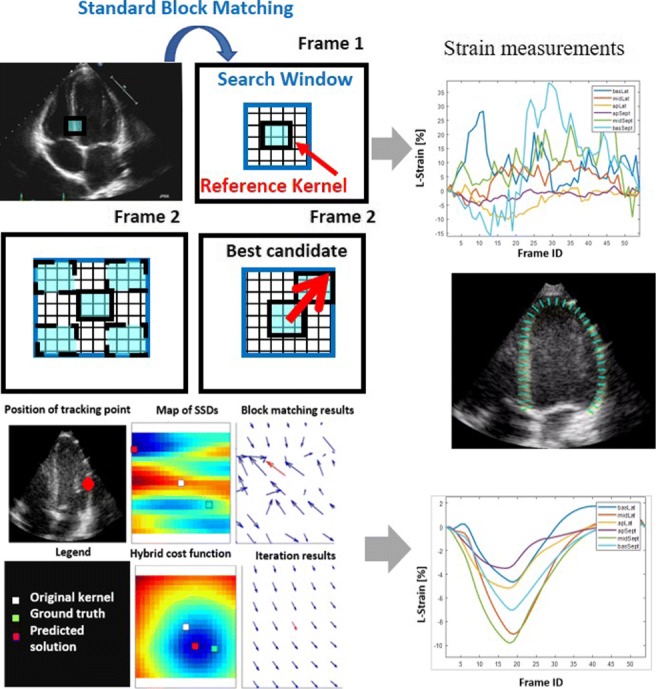
Standard block matching versus proposed iterative block matching approach

Standard block matching versus proposed iterative block matching approach

## Introduction

Cardiac ultrasound (echocardiography) is the most widely used imaging technique in cardiology. However, different commercially available software packages used in clinical practice yield unsatisfactorily wide discrepancies between measurements on the same patient images, wider even than 10% proposed as acceptable by the “task force” of clinicians and vendors [1]. In this study, we propose a method of improving resistance to image noise by applying a penalty for spatial inhomogeneity of velocity.

When cost is immaterial, techniques such as cardiac magnetic resonance imaging with semi-automated quantification software offer improved accuracy and precision, but this is not practical in everyday cardiology. New echocardiographic techniques, such as strain imaging, have emerged as promising quantitative tools in measuring left ventricle (LV) function with superior prognostic value to ejection fraction for predicting adverse cardiac events [2]. Clinical feasibility of strain resulting from speckle tracking echocardiography (STE) has been shown in many studies [3–10]. For example, strain has been used for the detection of myocardial ischaemia; it may apply after coronary reperfusion to predict infarct size; it is suggested for patients during chemotherapy to detect a decline in cardiac function early. Similarly, strain has been proposed to estimate the risk of ventricular arrhythmias; it may apply to find the optimal position for the pacing lead in the LV free wall in the evaluation of patients after implantation of cardiac resynchronisation therapy [11].

Although there are commercially available STE software packages, the measurements they provide are mutually inconsistent. To address this issue, the European Association of Cardiovascular Imaging (EACVI) and the American Society of Echocardiography (ASE) along with representatives from all vendors have been endorsing a “task force” aimed to reduce the inter-vendor variability of strain measurement. They propose acceptance in the clinical practice of inter-vendor variability up to 10% [1, 12]. However, currently used software packages have variability exceeding 10%.

Speckles are created when a random group of scatterers is illuminated by waves bearing a wavelength larger than the size of the individual scatterers. The speckle pattern remains approximately stable from frame to frame. Tracking these speckles frame by frame will allow the extraction of some parameters such as displacement and strain [13, 14]. For example, in a medical application such as evaluation of cardiac function, tracking these speckles and analysing them can help to quantify the myocardial function.

The processing of ultrasound images is difficult due to typically high levels of noise contained within them. For example, in cardiac ultrasound images, tracking walls of the heart is problematic, because of the high level of noise, the lower resolution in the lateral wall, and the nature of the heart motion. Different approaches for the speckle tracking in ultrasound sequences have been proposed, but it is a complicated task in which there is room for improvement [15–19].

Several models have received extensive attention in the ultrasound engineering community, such as block matching (BM) [20–23], optical flow [24–26], elastic registration [27, 28], and machine learning models [29, 30]. The most computationally efficient method of quantifying tissue motion on ultrasound is BM.

Traditional BM approaches are extremely vulnerable to the presence of image noise, which is always present in everyday clinical cine loops [1]. Since BM possesses conceptual simplicity and high computational speed and can provide a robust estimation of the motion by comparing the similarity between blocks of two images or two video frames, it has been commonly used in the ultrasound community [15, 31]. Several studies have attempted to improve the accuracy of the speckle tracking algorithms. Active shape models have been used to extract several physical properties of the myocardium in its different layers by applying some constraints to improve the accuracy of the motion estimation [32]. An optimisation process that integrates the physiological constraint of smoothness of the displacement field into the tracking algorithm to overcome the limitation of speckle decorrelation noise has been introduced [20]. The use of the Viterbi algorithm to overcome the effect of peak-hopping error has also been reported [33].

Inspired by the work of Khamis et al. [20], in this study, we have investigated the feasibility of speckle tracking in cardiac synthetic ultrasound sequences using an optimisation-based problem, which is solved iteratively.

## Methods

### Standard block matching

**Fig. 1:**
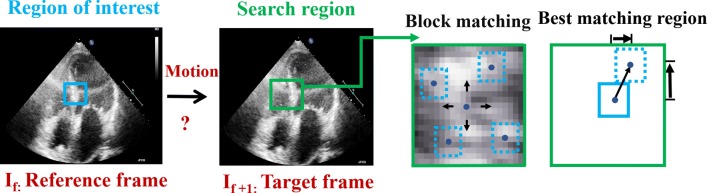
Speckle tracking using BM where a region in the image (kernel) is selected and sought for in the next image by sequentially trying out different positions, testing the similarity between the kernel and the pattern observed in that position. The position where the similarity between the kernel and the observed pattern is maximal is accepted as the new position of the original kernel

Classic BM begins by positioning a window on one frame and searching for a pattern with the most similar features within the dimensions of the placed window in the next frame. A cluster of speckles can be combined into one functional unit which is called a kernel; each kernel has a unique fingerprint that is determined using a similarity measure and can be tracked throughout the entire cine loop by the BM algorithm. In the reference frame (first image in Fig. 1, the current frame or a frame at time *t*_0_), the region of interest (blue square) has speckle patterns. In the next frame (a frame at time t + 1), a broad region of the image is searched for a similar speckle pattern. The location whose speckle pattern matches best is considered to be the estimated new location of the original kernel, thereby providing an estimated displacement vector.

This procedure is repeated across the whole of the reference frame, obtaining a displacement map between the two images. Repeating this procedure across the whole image sequence produces a vector field across space and time. In this study, sum of squared differences (SSD) is used as a similarity measure which calculates the difference between the intensity pattern of a grid of pixels (original kernel) in one frame and a set of identically sized kernels in the next frame, to find the best-matched kernel. Assuming a (m×n) kernel, the comparison between a kernel in the current reference frame (*I*_1_) and a kernel in the target frame (*I*_2_) moved by (p, q) pixel is

1 $$ SSD (p,q)=\sum\limits_{i=0}^{m}\sum\limits_{j=0}^{n}\left (I_{1}\left (i, j \right ) - I_{2} \left (i + p, j + q \right ) \right )^{2}  $$

where *p* and *q* are shift components along the x-direction and y-direction, respectively. The lowest SSD value indicates the most probable direction of the movement of the tissue. Effectively, the BM algorithm tracks the speckles by minimising a cost function. This method is based on the assumption that the SSD value should gradually increase as blocks move further away from the best-matched kernel. Since the smallest step size within the search window is one pixel, it is only able to evaluate the displacement vector to one pixel. To achieve sub-pixel accuracy, a parabolic fitting method was implemented [34].

To estimate the motion with sub-pixel precision in the spatial movement, two orthogonal parabolic curves were fitted to the horizontal and vertical of SSD values along with the best matching position. The local minima of the fitted curves were then selected as the final solution, which allows the displacement vector to be evaluated with sub-pixel precision. Based on the parabolic model, denoted by the Eq. 2 where a, b, and c are the real constant values, the minimum of the curve can be found by differentiating and setting the derivative to zero, as shown in the Eq. 3:

2 $$ yy=ax^{2}+bx+c  $$

3 $$ \frac{yy}{dx}=2ax+b=0\quad\quad\quad x= -\frac{b}{2a}  $$

Substituting the SSD values for each of these three-data points into the Eq. 2 will give

4 $$ P_{1}=a-b+c \quad\quad P_{2}=c \quad\quad P_{3}=a+b+c  $$

where *P*_2_ is the minimum SSD value from the kernel and *P*_1_ and *P*_3_ are SSD values from the neighbouring position on either side. The sub-pixel shift *x*_0_ was computed by

5 $$ _{X_{0}} = \frac{P_{1} - P_{3}}{2P_{1}-4P_{2} +2P_{3}}  $$

This was done for horizontal and vertical components separately, and the shift values were added to the corresponding integer displacements (*p* and *q* in Eq. 1) to obtain sub-pixel accuracy.

### Proposed optimised block matching approach

In this paper, a new displacement estimation method is introduced by formulating the tracking as an optimisation problem that jointly penalises intensity disparity and motion discontinuity and is, therefore, more robust to the signal decorrelation when compared with previous approaches. The speckle tracking algorithm combines the BM algorithm with a smoothness constraint for a neighbourhood of kernels, and minimises the following cost function:

6 $$ \text{Cost function} = \sum\limits_{r}(E_{SSD} + \lambda E_{N})  $$

where *r* is the total number of kernels being tracked and *E*_*SSD*_ is the sum of *r* SSD values. The second component of the cost function (${\sum }_{r}$*E*_*n*_) is a penalty function for speckle (i.e. intensity) decorrelation which penalises the motion discontinuity, and *λ* is the regularisation weight. This optimisation problem is then solved iteratively.

**Fig. 2:**
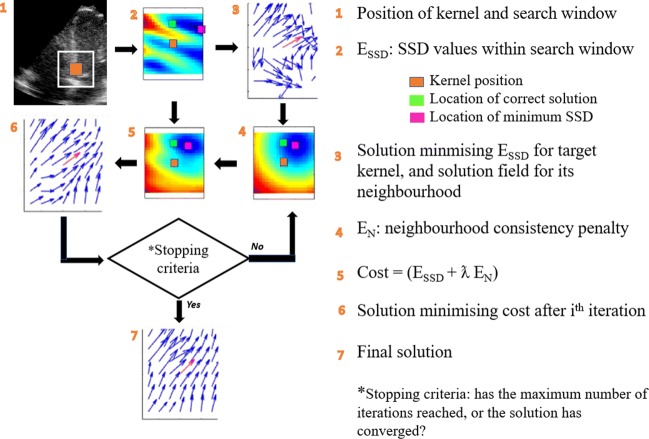
Flowchart showing the steps involved in solving the proposed optimisation-based tracking algorithm

For the first iteration of the tracking algorithm, the calculated displacement vector field will be smoothed by applying a median filter with kernel size identical to the neighbourhood size. An overall representative displacement vector for the neighbourhood is then obtained by taking the average of all kernels in the neighbourhood. Then, the difference between each potential position in the search window for a kernel and the representative vector is calculated. This is done for *r* kernels being tracked, and the sum will be the term ($\sum r En$) in Eq. 6. In the next stage, the overall cost function for each kernel’s candidate positions will be computed, incorporating the original SSD values and the penalty term obtained in the previous step. An updated displacement vector field will then be computed by taking each kernel’s candidate position with the lowest cost value, and the process is repeated by estimating a new average representative vector. After each iteration, the new displacement field will be used as an input to the next iteration until either no further changes are observed, indicating the optimisation problem is converged, or a maximum number of iterations is satisfied. Figure 2 provides an overall view of the working principles of the proposed tracking algorithm.

### Tracking parameters

The standard BM was carried out with a kernel size of (11×11) pixels with a spacing of 1 pixel, providing a dense solution. This kernel size is deemed to be a good compromise for the optimum tracking accuracy.

The size of a search window is also important since a small size would result in the algorithm failing to capture the larger displacements occurring between consecutive frames, and excessively large search window sizes would result in features outside the maximal feasible translation distance to be evaluated as possible links. An optimum size can be estimated from the maximum possible velocity of the myocardium, frame rate, and spatial resolution of the images (i.e. pixels per mm). However, due to the lack of such information being available about the synthetic sequences made accessible, we adopted the trial and error method to estimate a reasonable size for the search window, which turned out to be 21×21 pixels (central pixel ± 11 pixels). We then verified this adopted size by reviewing the ground-truth (maximum simulated displacement between any pair of frames) across all image sequences.

For the optimised BM approach, the number of iterations was set to 20, which was deemed to be a good compromise between the accuracy and computational run time; a threshold for which the solution was converged and any further update in the displacement vectors were insignificant. The parameter *λ* was 0.3, giving more emphasis to the data term versus the regularisation term in Eq. 6. Larger values of *λ* tend to heavily regularise the displacement vectors, which would result in an unrealistically uniform vector field where most of the vectors are aligned. A neighbourhood of (45×45) kernels was included in the iterations for updating the solution for the central kernel. The tracking accuracy was estimated by comparing the displacement field obtained from the speckle tracking algorithms and the ground-truth.

### Strain calculations

Strain describes the deformation of an object normalised to its original shape and size. Using the displacement vectors obtained from the speckle tracking, and according to the recent recommendations from the EACVI/ASE/Industry Task Force [1, 12], the Lagrangian strain was calculated as

7 $$ {\epsilon \left (t \right )}= \frac{L(t) - L_{0}}{L_{0}} $$

where *L*(*t*) is either the length of a segment (in case of segmental/regional strain) or the length of the LV contour (in case of global longitudinal strain (GLS)) at a given point in time, and *L*_0_ is the reference length at the reference time *t*_0_. In the case of computing GLS, *L*_0_ is the total longitudinal length of the LV border in end-diastole. The length was computed by using a continuous interpolation by cubic splines. Strain is a dimensionless entity, reported as a fraction or percent [35, 36]. Since we were interested in strain measurements in the LV only, the images were cropped manually before speckle tracking process, by considering a rectangle containing the LV. However, the initial positioning of the tracking kernels was automatic.

### Ultrasound dataset

The performance of the two speckle tracking approaches was evaluated using a publicly available synthetic cardiac dataset for which the ground-truth (exact solutions) are known [37]. Synthetic ultrasound images from 7 major vendors have been provided: GE, Hitachi-Aloka, Esaote, Philips, Samsung, Siemens, and Toshiba. The simulation process is briefly described here (further details can be found in [37]). The ultrasound images were simulated from a cloud of point scatterers (scatter map) and using an ultrasound simulator [38]. To take realistic speckle texture for each vendor, scattering amplitude was sampled from a 2D real clinical recording ultrasound as a template. Then, an electromechanical cardiac model was used to relocate the scatterers inside the myocardium and to have a realistic heart motion in the simulated images. Moreover, synthetic probe settings such as scan depth, focus depth, beam density, etc. were specialised by using the values communicated by each vendor upon signature of nondisclosure agreements.

The ground-truth has been provided as a set of seed points (36 points) along the longitudinal, and 5 points in radial directions. Points were further subdivided into six segments by splitting the endocardial contour into six parts with the same length as shown in Fig. 6 (top panel). Synthetic images were provided for normal (healthy) and ischaemic cases for each vendor. Only the apical 4-chamber (A4C) views, which is the most commonly used echo view, were included for longitudinal strain calculation. The total numbers of frames for vendors GE, Hitachi-Aloka, Esaote, Philips, Samsung, Siemens, and Toshiba, were 54, 72, 54, 50, 65, 56, and 60 respectively. Also, the image size for each vendor is as follows: GE, 479 × 616; Hitachi-Aloka, 565 × 811; ESAOTE, 580 × 682; Philips, 487 × 619; Samsung, 381 × 483; Siemens, 617 × 736; and Toshiba, 489 × 636. The pixel depth was 8 bit, providing an intensity resolution of 256 grey levels in the synthetic images.

## Results

### Displacement vector field

**Fig. 3:**
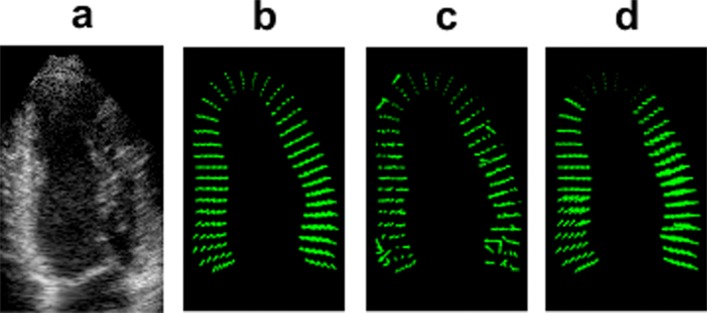
An example A4C from the Siemens healthy sequence and corresponding displacement vector fields during the rapid ejection phase (peak systole). **a** Zoomed view of LV cropped from the original image. **b** Ground-truth. **c**–**d** Displacement fields obtained from standard BM and optimised BM approach in the rapid ejection phase, respectively. Corresponding figures for other vendors are provided in Appendix A

The tracking parameters were similar for all vendors and cases. The algorithm returned a dense displacement field between pairs of consecutive frames. Figure 3 illustrates an example A4C view from the healthy Siemens sequence in the rapid ejection phase (peak systole), together with the corresponding ground-truth. The computed displacement vector field by the two tracking approaches (standard BM and optimised BM approach) is also shown. The presence of noise in the results is evident in the standard BM technique, whereas the optimised BM approach seems to suffer less from this problem.

**Fig. 4:**
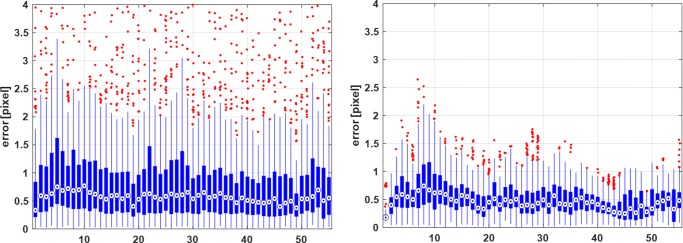
Boxplots of the error for the healthy sequence from Siemens. The error is computed as the magnitude of the difference between the calculated and ground-truth displacement vectors and is provided for standard (left) and proposed (right) tracking methods. The x-axis shows the frame number

**Fig. 5:**
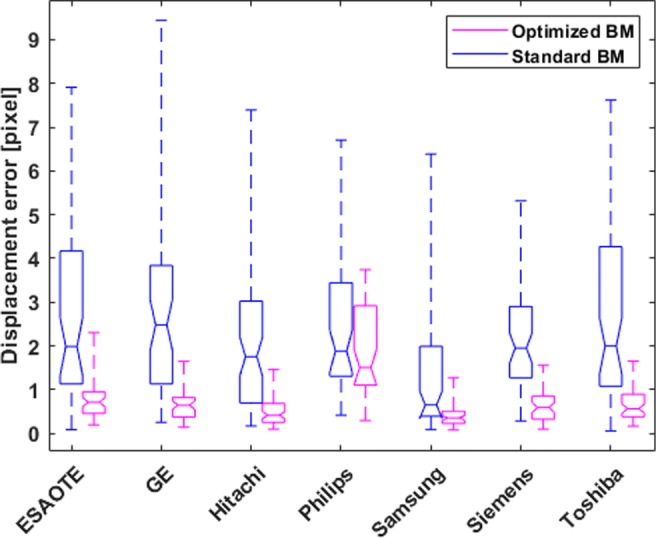
Displacement error for the healthy A4C synthetic sequences across all vendors for the two speckle tracking approaches. The horizontal line represents mean; the box signifies the quartiles, and the whiskers represent the 2.5% and 97.5% percentiles

Figure 4 shows the distribution of error for the same image sequence, obtained from both tracking methods. The displacement errors across all vendors for their corresponding healthy image sequences are shown in Fig. 5.

### Regional and global strain measurements

**Fig. 6:**
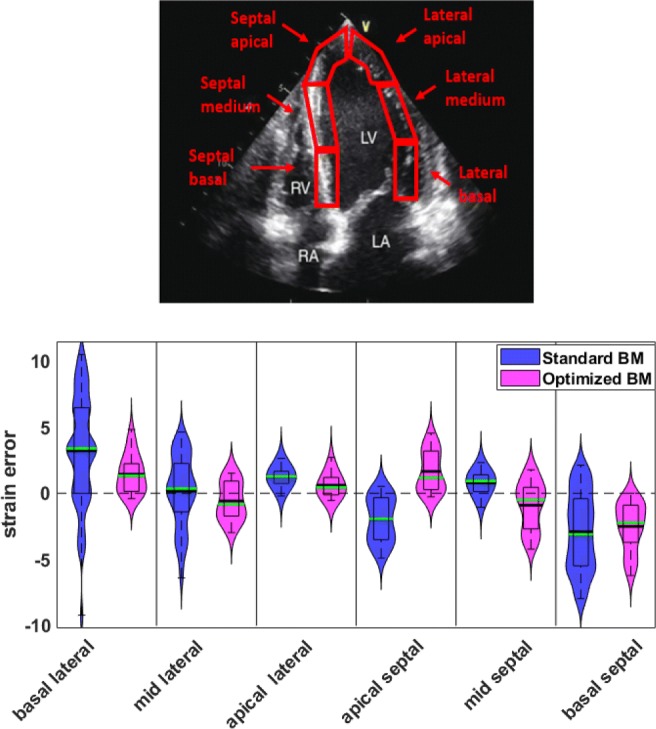
Top: an A4C view with the LV myocardium segmentation regions overlaid. Below: violin plots of the error in the segmental strain measurements for the healthy synthetic sequence from Siemens. The solid black line represents mean, and the green line represents the median; the box signifies the quartiles, and the whiskers represent the 2.5% and 97.5% percentiles

Regional (segmental) longitudinal strain values were calculated from the estimated displacement vector field. Figure 6 displays the violin plots of the regional strain error (the difference between the speckle tracked and the ground-truth) for all LV segments, for the same image sequence as shown in Figs. 3 and 4.

**Fig. 7:**
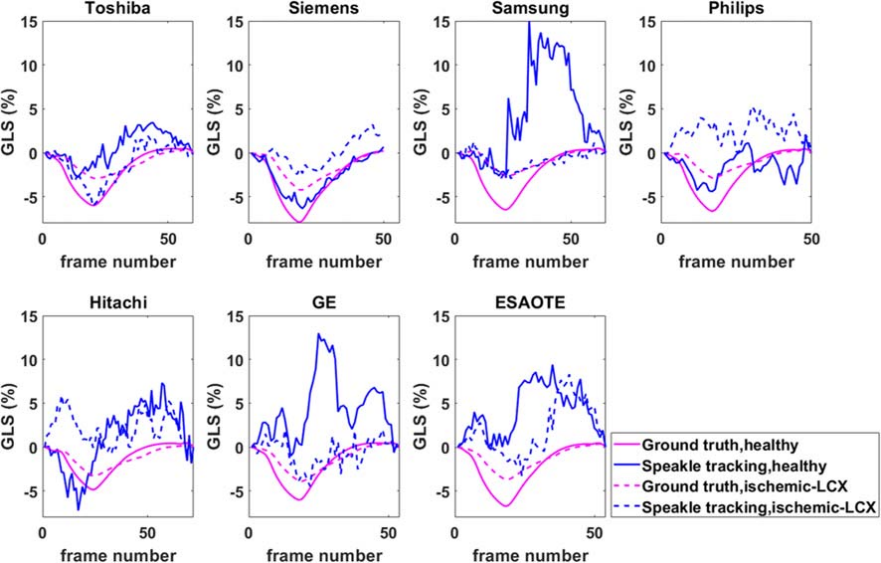
Comparison of GLS measurements obtained from the standard BM approach for the healthy and ischaemic LCX (ischaemic left circumflex coronary artery) cases across all vendors with the known ground-truth. The solid and dashed blue lines represent the calculated strain values for healthy and ischaemic cases, respectively. The solid and dashed magenta lines indicate the corresponding ground-truth

**Fig. 8:**
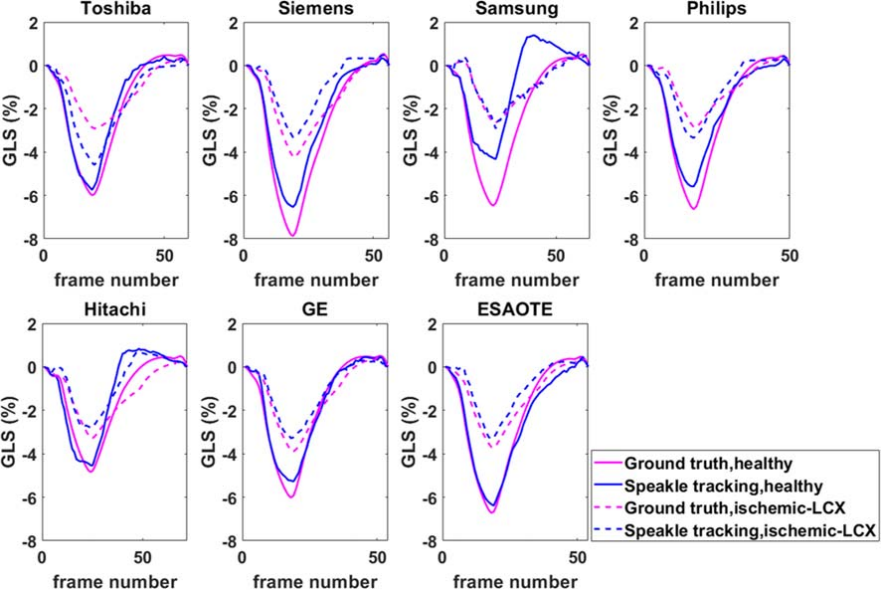
Same as Fig. 7, but for the optimised BM approach

GLS values were also computed from the estimated displacement vector fields for the healthy and ischaemic sequences across all vendors. The results are provided in Figs. 7 and 8 for the standard BM and the optimised BM approaches, respectively. An improvement in the case of the optimised BM approach is evident. The statistical analysis of standard and optimised BM approaches has been presented for the GLS measurements for the healthy and ischaemic cases across all vendors in Tables 1 and 2, respectively.

**Table 1:** Statistical analysis of standard and optimised BM approaches for the GLS measurements for healthy sequences across all vendors. The slope of the regression line (*α*), correlation coefficient (*ρ*), bias (*μ*), upper limits of agreement (ULOA), and lower limit of agreement (LLOA) are provided

	Standard BM					Optimised BM				
Vendor	*α*	*ρ*	*μ*	ULOA	LLOA	*α*	*ρ*	*μ*	ULOA	LLOA
Hitachi	0.28	0.57	2.34	8.05	–3.36	0.86	0.93	0.29	1.61	–1.03
Toshiba	0.93	0.72	2.42	5.48	–0.63	1.05	0.98	0.20	0.91	–0.50
Esaote	0.20	0.23	6.17	12.62	–0.27	1.08	0.99	− 0.17	0.51	–0.86
Samsung	0.14	0.33	6.15	16.02	–3.71	1.09	0.89	1.20	3.34	–0.93
Siemens	1.27	0.97	0.40	2.02	–1.21	1.18	0.99	0.48	1.44	–0.46
Philips	0.81	0.51	0.55	4.63	–3.53	1.21	0.99	0.19	1.12	–0.72
GE	0.04	0.08	5.96	14.10	–2.16	1.07	0.99	0.05	0.61	–0.49

**Table 2:** As Table 1, but for ischaemic sequences

	Standard BM					Optimised BM				
vendor	*α*	*ρ*	*μ*	ULOA	LLOA	*α*	*ρ*	*μ*	ULOA	LLOA
Hitachi	0.06	0.18	1.82	10.20	–6.55	0.69	0.74	–0.12	1.96	–2.21
Toshiba	0.28	0.49	1.74	6.34	–2.84	0.84	0.97	–0.26	0.64	–1.17
Esaote	0.18	0.28	3.77	8.96	–1.42	1.07	0.97	0.35	1.15	–0.43
Samsung	0.26	0.43	0.63	5.13	–3.87	0.90	0.86	0.54	2.07	–0.99
Siemens	0.73	0.66	0.74	3.38	–1.88	1.08	0.95	0.47	1.56	–0.60
Philips	0.02	0.32	0.48	40.75	–39.79	0.02	0.34	–1.89	31.98	–35.77
GE	–0.07	–0.14	2.15	9.15	–4.85	1.05	0.98	0.29	0.89	–0.30

## Discussions

As can be seen in Fig. 4, the optimised BM approach suffers less from the presence of outliers and noise spikes in the computed displacement field. The significant errors in standard BM appear to correspond to the cardiac phases when the heart muscle has the highest velocities, which happen during the rapid ejection phase. For such instances, the magnitude of the displacement is high, and the deformations are relatively large, resulting in lower SSD peaks (or other similarity measures such as correlation coefficient) in BM. Therefore, secondary peaks caused by random correlations between speckle kernels can sometimes exceed the actual peak. This effect can produce “peak-hopping” artefacts in which a secondary peak is chosen as the best match within a search region, giving rise to significant errors in displacement and deformation estimates. However, the optimised BM approach seems to be less prone to this phenomenon as the fidelity of the solution is checked by the neighbourhood consensus representing the overall motion of the myocardium.

The optimised BM approach demonstrates consistently lower errors across all vendors except Philips (Fig. 5). In the case of synthetic sequences from Philips, the two tracking approaches behave similarly, with the optimised BM approach performing slightly better.

Similar behaviour is observed in the calculated strain measurements. A considerable improvement in the basal segments (both lateral and septal) can be seen in the optimised BM approach when compared with the standard BM approach. This is likely to be because of the fast-moving heart muscles in these segments for which the standard BM struggles to track, most likely due to the peak-hopping artefacts. For the apical segments, where the site of measurement is in the vicinity of the apex and, therefore, moves at relatively lower velocities, the error in both methods drops, relative to the corresponding basal segments.

Overall, the optimised BM approach demonstrated better performance in estimating the GLS values in comparison with the standard BM (Tables 1 and 2). In case of ischaemic GE sequences, a close to zero correlation coefficient for the standard BM indicated very poor tracking results, where the optimised approach seems to be offering more reliable results, with a correlation coefficient of 0.98. For the ischaemic sequences from Philips, however, both tracking approaches suffer from poor strain measurement errors, with correlation coefficients of 0.32 and 0.34, respectively. The simulated image sequences for both vendors have relatively poorer image qualities, with segments of the myocardium is missing/invisible in the simulated imaging plane, where the tracking algorithms struggle to follow the speckle movements between consecutive images. For all other vendors, the optimised BM approach demonstrates an acceptable level of accuracy.

Considerable biases are observed in both healthy and ischaemic cases in GLS values obtained from the standard BM approach and for some of the vendors (Fig. 7). It should be noted that such poor results are less likely to be observed when using vendors’ software packages. This is because here we have presented the results from a purely BM step where no additional post-processing is applied. From a clinical image sequence, speckle matching alone never provides an unambiguous, obviously correct, velocity field. Physical limitations of ultrasound, and out-of-plane motion, prevent perfect speckle matching. There are often several ways that a speckle pattern in one frame could transform into its counterpart in the next frame.

**Fig. 9:**
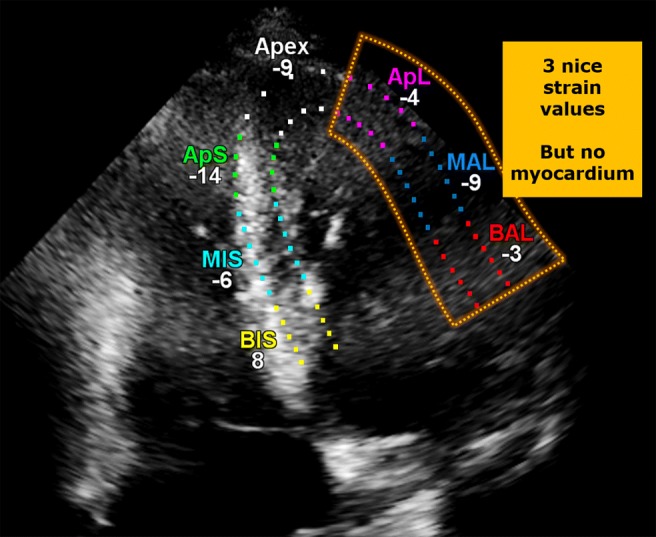
Example of a (presumably) “common sense” editing in a commercial package on one frame, where 3 regional strain values are given for the lateral wall, but there is no visible myocardium to be tracked in that region

Therefore, we presume the current strategy undertaken by most vendors is a 2-step process. First, calculate the displacement vector field maximising the match between successive frames (i.e. standard BM). Second, apply automated “common sense” editing that weeds out implausible vectors, and instead infer values using regions adjacent in space and/or time (i.e. spatial or temporal filtering). Figure 9 illustrates an example providing evidence for this “common sense” editing, where 3 reasonable strain values are given for the lateral wall, but there is no myocardium to be tracked in that region.

Exactly how this “common sense” works has a large effect on all downstream results including strain. This can potentially explain the persistent contradiction between vendors despite their standardising definitions for the acquisition and nomenclature. Task force acknowledges the inability to resolve the vendor discrepancy and recommends follow-up measurements to be done with the same software as before. This causes logistical problems (if a hospital has > 1 vendor) or vendor lock-in [39].

Interestingly, a recent study [40] has concluded that post-processing is the most important determinant in inter-vendor variation, with differences in acquisition having a small effect. None of the vendors included in this study has disclosed its algorithms for strain measurements. Therefore, could not reproduce the result of their corresponding software packages for a direct comparison here.

Speckle decorrelation is signal- and motion-dependent. Therefore, it cannot be compensated for by simple post-tracking spatial or temporal smoothing. Thus, the proposed approach, simultaneously maximises match and penalises implausibility (fusing BM and biological constraints), optimised by minimising the two-element cost function. The optimisation process jointly maximises signal correlation and motion continuity (we give an analogy in Appendix B), eliminating the need for subsequent editing of the raw displacement vectors which is probably the underlying cause of vendor discrepancy.

Figure 8 illustrates that by minimising the cost function in Eq. 6, the estimated strain values are more reliable for both healthy and ischaemic cases and across all vendor except the healthy case for the Samsung image sequences, where a considerable bias between the calculated strain and the ground-truth is evident. This is most likely due to the missing/smeared walls in the images as shown in Appendix A, where the algorithms fail to return meaningful speckle-tracked displacement vectors.

We have implemented our algorithms in C++ programming language, and it currently takes a maximum of 10s to process a pair of frames using an Intel Xeon E5630 CPU, with an internal clock frequency of 2.53 GHz. Our follow-up studies will look into the implementation of the algorithms on the graphics processing units for parallel computations, from which we are expecting > 1000-fold increase in the processing speed. This should make the run time feasible for the offline use in the clinical practice, or even a real-time application.

## Study limitations

Our study considered only the A4C view, which is the most common apical probe orientation. However, no view-specific assumptions were made during the algorithm developments, and the proposed tracking method should, in principle, be applicable to other echo views. Therefore, future studies would include other standard echocardiographic views such as 2-chamber and 3-chamber. Additionally, only synthetic image sequences were used for evaluating the performance of the tracking algorithms. This provided the advantage of knowing the exact solution (ground-truth) for the speckle tracking which could be used for error calculations. However, an immediate follow-up study will consider using patient echocardiographic image sequences, representing real-world clinical data.

The purpose of this study was to examine the performance of an improved speckle tracking technique in estimating the displacement of strain measurements. Hopefully, this would serve as a stepping stone to addressing the issue of inter-vendor variability, which has become the main limitation to the implementation of this technique in clinical settings. Assuming the vendor discrepancy is partly due to different “common sense” editing and filtering techniques applied by the vendors to the erroneous speckle tracking results (to make the results see more reasonable), this improved version of tracking could potentially help in reducing the variability by eliminating the need for all subsequent editing of the results. A thorough investigation of this issue would require the use of echocardiograms obtained from the same patient, but using different vendors. The dataset available and used in our study provides sequences from different vendors and patients. Therefore, a direct comparison of the results to examine the inter-vendor variability was not possible in the current study. A future comprehensive study must examine the potential influence of the proposed tracking algorithm on the inter-vendor variability in the strain measurements.

## Conclusion

An optimised-based speckle tracking echocardiography algorithm was proposed in this study. Its performance was evaluated using a publicly available synthetic echocardiographic dataset with known ground-truth. The results showed improved performance compared with the standard BM in estimating the displacement vector and longitudinal strain measurements. The proposed tracking method does not require any post-processing or filtering steps and can potentially reduce the variability in strain measurements caused by various implementations of such filtering techniques.

## Appendix A

An example A4C from 6 vendors with the healthy sequence and corresponding displacement vector fields: (a) zoomed view of LV cropped from the original image, (b) ground-truth, (c)–(d) displacement fields obtained from standard BM and optimised BM approach in the rapid ejection phase, respectively.

## Appendix B

What is different in the proposed approach is that it takes into account both BM and regularisation simultaneously. This is not a trivial difference.

A very simplified analogy would be traversal of the maze shown opposite (upper panel). Imagine an algorithm was to “optimise the X coordinate” (middle panel) followed by “optimise the Y coordinate” (lower panel). This would not be able to solve the maze. Even if the algorithm was to alternate between optimising X and Y repeatedly, it would not be possible to solve the maze by optimising each alone. This is because an apparent improvement in one parameter, e.g. the y-coordinate becoming closer to the y-coordinate of the exit of the maze, may actually be moving further away from solving the maze because it leads to a dead end.

The best way to solve the maze is to consider the entire search space of X and Y. It is this consideration of both parameters simultaneously, rather than one after the other, which the proposed approach provides.

More formally, the major difference between our approach and a classic (standard) BM approach is the way the solution is obtained by minimising the cost function. In the classic approach, a best match solution such as minimum sum of squared differences (or other similarity measures) is obtained first, and the results are then smoothed by applying regularisation constraints. The two-stage procedure does deliver a local smoothing of displacement vector field, but when the best match is discarded, it is replaced by interpolated values from neighbours rather than by going back to the original image to find a displacement vector that is not only physiologically feasible (by correspondence to neighbours) but visually plausible. Therefore, it does not fully use the information available.
